# In-airway molecular flow sensing: A new technology for continuous, noninvasive monitoring of oxygen consumption in critical care

**DOI:** 10.1126/sciadv.1600560

**Published:** 2016-08-10

**Authors:** Luca Ciaffoni, David P. O’Neill, John H. Couper, Grant A. D. Ritchie, Gus Hancock, Peter A. Robbins

**Affiliations:** 1 Department of Chemistry, Physical and Theoretical Chemistry Laboratory, University of Oxford, South Parks Road, Oxford OX1 3QZ, UK.; 2 Department of Physiology, Anatomy and Genetics, University of Oxford, Sherrington Building, Parks Road, Oxford OX1 3PT, UK.

**Keywords:** Gas lasers, respiration, oxygen consumption, gas exchange, medical device, medical technology, absorbence spectroscopy, laser spectroscopy

## Abstract

There are no satisfactory methods for monitoring oxygen consumption in critical care. To address this, we adapted laser absorption spectroscopy to provide measurements of O_2_, CO_2_, and water vapor within the airway every 10 ms. The analyzer is integrated within a novel respiratory flow meter that is an order of magnitude more precise than other flow meters. Such precision, coupled with the accurate alignment of gas concentrations with respiratory flow, makes possible the determination of O_2_ consumption by direct integration over time of the product of O_2_ concentration and flow. The precision is illustrated by integrating the balance gas (N_2_ plus Ar) flow and showing that this exchange was near zero. Measured O_2_ consumption changed by <5% between air and O_2_ breathing. Clinical capability was illustrated by recording O_2_ consumption during an aortic aneurysm repair. This device now makes easy, accurate, and noninvasive measurement of O_2_ consumption for intubated patients in critical care possible.

## Teaser

Laser absorption spectroscopy of respired gas offers new opportunities for monitoring critically ill patients.

## INTRODUCTION

Molecular oxygen acts as the terminal electron acceptor for oxidative metabolism, and thus, it is vital to metabolic function for all aerobic organisms. In critical care, this process of producing adenosine-5′-triphosphate (ATP) by oxidative phosphorylation may be compromised, and consequently, the measurement of the rate of oxygen consumption (${\dot{V}}_{O_{2}}$) may potentially be useful. However, current techniques for determining ${\dot{V}}_{O_{2}}$ in critical care are inadequate because they are invasive, are of limited accuracy, and typically only provide intermittent measurements. All attempts at noninvasive measurement have so far failed (*1*). ${\dot{V}}_{O_{2}}$ is the difference between the quantity of O_2_ breathed in and the quantity of O_2_ breathed out per unit time. The difficulty of using this to measure ${\dot{V}}_{O_{2}}$ noninvasively is that small errors in determining the amount of O_2_ breathed in relative to the amount breathed out give rise to large errors in ${\dot{V}}_{O_{2}}$. The problem becomes more acute when the inspired O_2_ fraction is increased, as is common in critical care. One technique for reducing this error, known as the Haldane transformation, is to infer the amount of oxygen breathed in from only the expiratory measurements and hence calculate ${\dot{V}}_{O_{2}}$. The Haldane transformation assumes that, over a reasonable period, the net N_2_ exchange at the mouth is zero. Thus, if the inspired fraction of N_2_ is known and the total amount of N_2_ in the expirate is measured, then the inspired volume can be calculated directly without measurement. The advantage of this approach is that small errors in the expiratory measurements lead to “compensatory” small errors in the volume of oxygen breathed in, which in turn limit the error in the estimate for ${\dot{V}}_{O_{2}}$. The calculation requires that the O_2_ and N_2_ fractions of the inspired gas are known. Thus, it works well with air as the inspired gas—for example, measurements of ${\dot{V}}_{O_{2}}$ during cardiopulmonary exercise testing that use this technique. However, it cannot be used for critical care in which the inspired O_2_ is typically elevated and varies over time.

The purpose of this study was to develop new technology for measuring gas exchange at the mouth with sufficient precision to render the use of the Haldane transformation unnecessary. Hence, it should provide a technique for monitoring ${\dot{V}}_{O_{2}}$ in critical care that will work regardless of whatever the fraction of O_2_ in the inspired gas is. Mathematically, the problem is equivalent to evaluating the integral

$${\dot{V}}_{O_{2}}=\frac{1}{\Delta t}\int_{t}^{t+\Delta t}\dot{V}(t)\cdot F_{O_{2}}(t)dt$$ (1)

where Δ*t* is the period of integration and $\dot{V}(t)$ and $F_{O_{2}}(t)$ are the respiratory flow rate and fractional concentration of oxygen at time *t*, respectively. To achieve the precision required, it is necessary to ensure that the instantaneous flows and concentrations in Eq. 1 are accurately time-aligned and that both measurements are made using techniques that have sufficient precision and sufficiently rapid dynamic responses to follow the changes accurately during a breathing cycle.

**Fig. 1:**
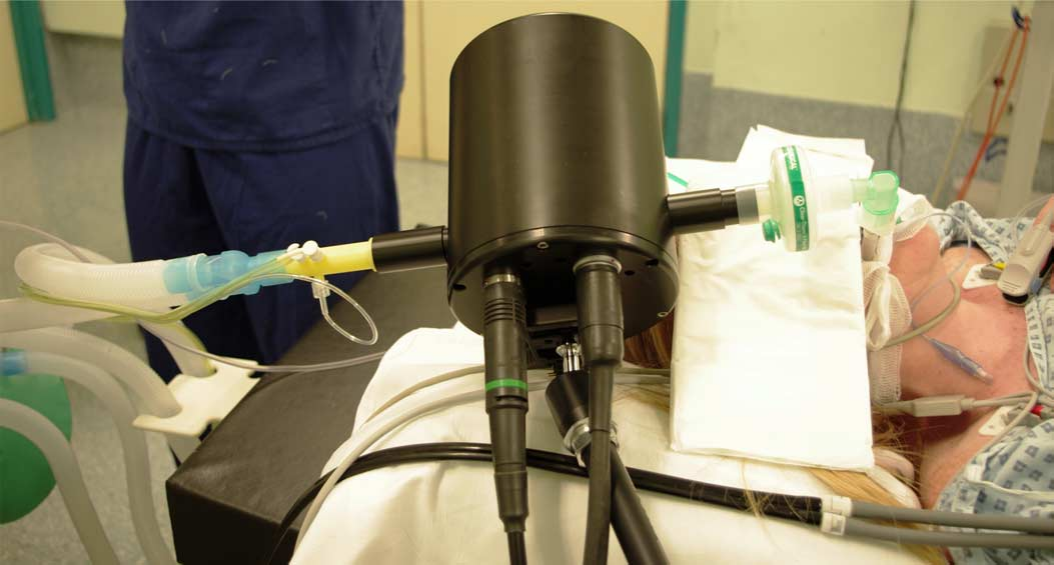
MFS in use during surgery. MFS measurement head incorporated in a closed-loop anesthetic delivery system. A 5-m-long pair of cables connects the measurement head to the control unit located away from the operating table.

To address this challenge, we first developed the highly accurate technique of laser absorption spectroscopy for the analysis of oxygen, carbon dioxide, and water vapor concentrations directly within the main airway every 10 ms. Unlike traditional sidestream gas analyzers that rely on a small portion of the respired gas being diverted to the measuring site through a sampling catheter, this mainstream arrangement ensures that the measurements of gas concentrations are precisely aligned in time with measurements of respiratory flow. Second, we developed a precision flow meter based on the principles associated with pneumotachograph-type devices. Here, we introduced a number of new features to its construction and calibration, which, together, achieve an order of magnitude improvement in precision over that currently specified in guidelines for such measurements (*2*). One particularly important feature is that the contemporaneous gas analysis enables dynamic calibration of the flow meter for the changes in density and viscosity that occur throughout the respiratory cycle. We demonstrate the performance of this instrument [which we term molecular flow sensor (MFS)] following a wash-in and subsequent wash-out of pure oxygen in a healthy volunteer. In addition to measuring ${\dot{V}}_{O_{2}}$, the device also calculates the exchange for CO_2_, water vapor, and the balance gas, which is determined by subtraction. Hereafter, we refer to the balance gas exchange as the N_2_ exchange, but this term includes other inert gases present, such as Ar. The balance gas provides a check on overall performance because N_2_ exchange should be very close to zero. We also illustrate a record of oxygen consumption obtained during general anesthesia with an elevated fraction of inspired oxygen. Figure 1 shows the MFS that is incorporated into a closed-loop anesthetic delivery system and connected to a mechanically ventilated patient.

## RESULTS

The details of the two novel features of the MFS, namely, the spectroscopic gas analyzer and the improved pneumotachograph, are given in Materials and Methods; this section (together with information presented in the Supplementary Materials) shows the tests that were carried out on the physical performance of the MFS. A summary of those results is presented in Table 1 and compared with recent guideline specifications recommended by the American Thoracic Society (ATS) and the European Respiratory Society (ERS) (*2*). Note that the volume accuracy is an order of magnitude better than the ATS/ERS guideline and that the synchronization of flow and gas analysis signals is within their target—something that no sidestream analyzer could reasonably deliver.

**Table 1:** Summary of MFS performance specifications. Comparison between MFS performance specifications and the guideline values recommended by the ATS and the ERS (*2*). NA, not available.

**Component**	**Recommended**	**MFS**
Volume accuracy	±3%	±0.2%
Sample flow	<20 ml/min	0 ml/min
Gas analyzer accuracy*	±1% FSO for O_2_	±0.45% FSO for O_2_
	±1% FSO for CO_2_	±0.5% FSO for CO_2_
	NA	±1.7% FSO for H_2_O
Gas analyzer precision^†^	NA for O_2_	±0.18% at 100% O_2_
		±0.39% at 21% O_2_
	NA for CO_2_	±0.20% at 8% CO_2_
	NA for H_2_O	±1.72% at 4.7% H_2_O
Gas analyzer, 10 to 90% rise time	<100 ms	10 ms
Data sampling frequency	≥100 Hz	100 Hz
Synchronization of flow and gas analysis	10 ms	5 ms

*Accuracy is defined as the maximum expected linearity error expressed as a percentage of full-scale output (FSO) range. We have taken the FSO as 0 to 100% for oxygen, 0 to 10% for carbon dioxide, and 0 to 6% for water vapor, with the upper limits chosen as the maximum percentage level of each gas expected in the respired mixture under the experimental conditions.

**Fig. 2:**
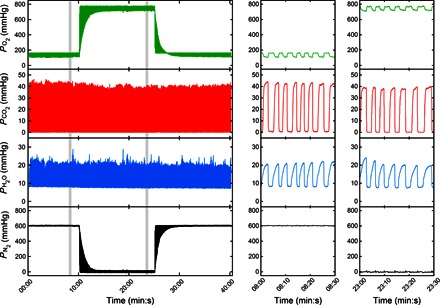
Partial pressures for the airway gases during an O_2_ wash-in and wash-out experiment. A volunteer was initially asked to breathe laboratory air flowing pass the exhaust side of the MFS through a Y-piece connector. The gas mixture was delivered at approximately 100 liters/min to ensure that no rebreathing of expired gas would take place. After 10 min of stable breathing, the inspired gas was switched from air to medical-grade O_2_ (>99.5%). Approximately 15 min were allowed for the nitrogen to be fully washed out from the lungs, and then the gas supply was switched back to air. Right panels show expanded versions of the data at times indicated in the main panel to illustrate intrabreath morphology.

†Precision is reported as coefficient of variation (CV), which is defined as the ratio of the SD (σ) to the mean (μ), expressed as a percentage. For each reference mixture, σ and μ are computed from 3 s worth of consecutive concentration values returned every 10 ms. For each channel, precision is reported at the highest concentration point of the calibration curve (see Fig. 2). For oxygen, the value estimated at 21% level is also stated.

### Performance of integrated system—Oxygen wash-in and wash-out

Here, we describe the tests conducted on human participants. The study received its ethics approval from the Scotland A Research Ethics Committee. All human studies were performed in accordance with international standards as set out in the Declaration of Helsinki, and informed consent was obtained from all volunteers before their participation in the study.

The performance of the overall system was examined in a healthy volunteer who undertook 10 min of breathing air, followed by 15 min of breathing pure O_2_, and then by a final 15 min of breathing air again. Pure O_2_ was chosen as the gas that provides the greatest challenge for accurate measurement of ${\dot{V}}_{O_{2}}$ because its concentration changes by only ~6% during a pure O_2_ breathing cycle. Figure 2 shows the airway partial pressures for the three measured gases together with the airway partial pressure for the balance gas (N_2_). Short sections during both air and pure O_2_ breathing are also shown on an expanded time base to illustrate the intrabreath morphology of the waveforms. As expected, the partial pressure for the balance gas (N_2_) is approximately zero during sustained O_2_ breathing. With reference to the water vapor trace, we are unaware of any previous recordings of water vapor throughout a respiratory cycle.

**Fig. 3:**
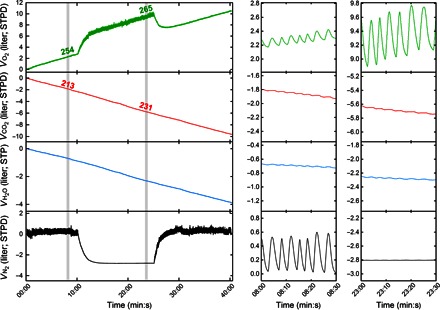
Results from direct integration over time to calculate cumulative gas exchange during an O_2_ wash-in and wash-out experiment. Left panel shows the data over the whole time period, and expanded time scales are shown in the two panels to the right. The slopes give the rate of gas uptake (negative in the case of CO_2_ and H_2_O). Values are given in ml/min above the shaded regions. Right panels show the scaling of the ordinate axes that have been kept the same to illustrate the relative contributions of each gas species to tidal volume. Note that, apart from the periods of washing N_2_ out of or into the lung, the overall slope of the N_2_ volume plot is close to zero, illustrating the precision of the measurements. STPD, standard temperature and pressure, dry.

Figure 3 illustrates the direct time integration of the product of instantaneous fractional abundance of each gas and the instantaneous flow. The slope of the cumulative volume plots (the quantity given in Eq. 1) indicates the net uptake (positive slope) or output (negative slope) of each gas species. Short sections are shown during both air and pure O_2_ breathing to illustrate intrabreath morphology. As expected, the largest excursions are for N_2_ during air breathing and for O_2_ during pure O_2_ breathing. There is no “gold standard” against which these results can be compared, but the N_2_ flux provides an internal calibration, particularly during air breathing, because this should be very close to zero. Note that even after 40 min of breathing (25 min of breathing air), following our measure of the integration error, the overall N_2_ exchange is essentially zero (<10 ml/min).

The effect of introducing small errors of ±3% into the inspiratory and expiratory flow calibrations is shown in the Supplementary Materials. The values of ±3% were chosen because these were the target precision specified in a consensus statement on respiratory flow measurement (*2*). We conclude that these errors lead to physically impossible values for respiratory exchange, particularly at high oxygen concentrations, illustrating the requirement for high precision in the instrument to make meaningful measurements.

### Performance of integrated system—Clinical deployment

**Fig. 4:**
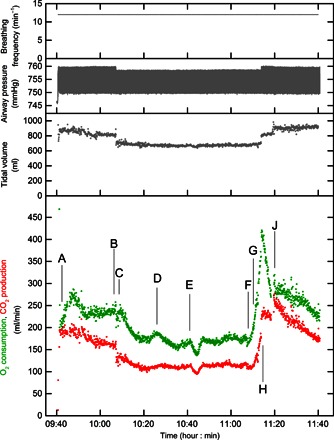
Gas exchange measurements during repair of an abdominal aortic aneurysm. Breathing frequency, airway pressure, tidal volume, oxygen consumption, and carbon dioxide production during elective repair of an abdominal aortic aneurysm. Oxygen consumption and carbon dioxide production are shown breath-by-breath, rather than as the continuous integral of Fig. 3. Events are represented by points as follows: (A) knife to skin; (B) reduction in ventilator driving pressure; (C) aortic clamp applied; (D) fall in blood pressure; (E) metaraminol (fast-acting α-agonist) bolus, and infusion rate increased from 2 to 5 ml/hour; (F) and (G) sequential removal of iliac artery clamps; (H) increase in ventilator driving pressure; and (J) removal of superior retractor restricting rib cage movement.

Figure 4 illustrates the use of the MFS in a mechanically ventilated patient; in this case, it is during the repair of an abdominal aortic aneurysm under total intravenous anesthesia. The inspired fraction for O_2_ is maintained at ~0.5 throughout. The record illustrates that, even when calculating oxygen consumption on a breath-by-breath basis, the values are extremely repeatable from one breath to the next. There is an obvious fall in O_2_ consumption when the aortic clamp is applied at point C and an equally obvious rise in O_2_ consumption when the iliac artery clamps are removed at points F and G. Also note the effect of a bolus/change in infusion rate of metaraminol at point E. Metaraminol is an α-agonist. Its vasoconstrictor properties will reduce perfusion to vascular beds, thereby reducing cardiac output and hence the rate of oxygen uptake within the lungs.

## DISCUSSION

The principal aim of this work was to develop a technology that enables accurate, simple, and noninvasive monitoring of oxygen consumption under conditions in which the inspired oxygen fraction is increased, such as occurs routinely in the setting of critical care. This required two major technical innovations. The first was to develop a method for rapid gas analysis within the main airway using laser absorption spectroscopy. An early proof-of-principle study that used free-space lasers on an optical bench suggested that this may be possible for CO_2_ and O_2_ (*3*). This report now describes a highly accurate device that can be used in a clinical setting. The second technical innovation was to provide a substantial improvement in the precision of pneumotachography.

Laser absorption spectroscopy is based on highly accurate physical principles, and hence, it is probably more accurate than any other form of respiratory gas analysis now available. Our ability to assess the technique’s accuracy is limited by the accuracy of commercial calibration mixtures, even when prepared using a gravimetric approach. As discussed in Results, the most valuable benefits of this gas sensing method over existing methodologies are the improved dynamic response and the elimination of transit delays associated with sampling catheters. The substantial advantages of the instrument are presented in Results, where its performance was compared with the desired specifications for respiratory gas analysis published in a relatively recent consensus statement between the ERS and the ATS (*2*). The respiratory mass spectrometer is recognized by some as “the current gold standard gas analyzer offering simultaneous measurement of multiple gases in constant compositions, full linearity, low sample flow, and short response time” (*4*). Compared with the mass spectrometer, our inline gas analyzer has no catheter delay (mass spectrometer, >200 ms), has a much faster response time (10 ms versus ~80 ms), and is capable of measuring water vapor. Furthermore, the mass spectrometer is expensive to construct and maintain because of its high vacuum system, whereas all components in our system are potentially cheap to manufacture in bulk and have no moving parts.

The use of pneumotachography to measure respiratory flow is a long-standing technique, and hence, the problems associated with it are all relatively well known. First, the relationship between the pressure drop and flow is not entirely linear and may depend on upstream geometry (*5*). Second, the relationship between pressure and flow depends on the physical characteristics of the gases, such that, for example, correction factors are required for different oxygen concentrations or some continuous analysis of respired gas composition is required to correct the flow measurement (*6*, *7*). Third, the presence of warm expired gas saturated with water vapor requires some element of heating to prevent condensation and subsequent alteration of resistance of the flow meter. The heating of the flow meter adds to the inaccuracies of flow measurement (*8*, *9*). Fourth, differential pressure transducers exhibit some degree of drift, which causes inaccuracies in the measurement of zero flow (*10*). Fifth, accurate flow standards are not readily available, and calibration has to proceed through the use of standard volumes delivered by calibration syringes (*11*, *12*). Each of these problems is addressed systematically in the present study, with the result that the accuracy of the technique is increased by approximately an order of magnitude.

There are some limitations to the current device; particularly, the head is rather large (height, 176 mm; diameter, 142 mm) and heavy (mass, 1.9 kg) and therefore needs supporting by some form of arm or strut. Ideally, the size and weight would be addressed in any commercialization of the device. One possibility is to move the oxygen laser from the head into the electronics module. Other possibilities include revising the construction of the housing and reducing the weight of the airway, which, in the present incarnation, is milled from a solid alloy block. The dead space of the device is currently 80 ml and, ideally, should be reduced. In a revision, it would be possible to achieve this by redesigning the light traps in the airway, which prevent any ambient light from entering the measurement cell.

The target applications of this device are for the measurement of oxygen consumption in mechanically ventilated patients. One potential application relates to the management of shock, which is a clinical syndrome that arises from many different causes but in which the common unifying feature is a failure to deliver and/or use adequate amounts of oxygen (*13*). Therapy is both supportive and directed toward the underlying cause, but overzealous resuscitation (for example, excessive use of inotropes or fluids) may itself be harmful. In septic shock, three recent major multicenter trials of target-based resuscitation have failed to improve outcomes over conventional treatment (*14*–*16*). One possible reason for this is the inadequacy of the targets, and, particularly, the absence of a fundamental measurement of how much oxygen is being consumed. Our device would make it possible to titrate therapies against their real-time effects on cellular oxygen utilization in individual patients.

Another potential application for the device is in the assessment of lung function. Conventional lung function tests provide clinical assessments for restrictive and obstructive lung diseases through the measurement of volumes and flows and also for limitations to diffusion through measurement of the diffusing capacity for carbon monoxide. However, classical lung function testing does not provide measurements for inhomogeneity of ventilation or perfusion within the lung. To address this, there is a growing interest in both lung wash-out studies and indices of inhomogeneous ventilation; one such index is the lung clearance index (*2*). However, a real limitation is the lack of precision with which these studies can be conducted, and this led to a consensus statement identifying the need for better technologies (*2*). Although the aim of our work was to provide measurements of oxygen consumption in critical care, the result was the development of an instrument that either meets or exceeds all the desiderata listed for lung wash-out studies.

A further potential application for this technology is for the measurement of oxygen consumption and carbon dioxide production in cardiopulmonary exercise testing. The ATS and the American College of Chest Physicians have provided a statement on cardiopulmonary exercise testing (*17*). In this statement, they recognize the attractiveness of a breath-by-breath methodology (one that does not use nitrogen balance and the Haldane transformation) for measuring pulmonary gas exchange, but they also recognize the considerable potential for erroneous data with this approach. The device presented here addresses the major causes of these erroneous data, namely, inadequate alignment of respiratory gas analysis with the flow signal caused by variable sampling catheter delays and insufficiently accurate measurements of respiratory flow. Breath-to-breath methodology such as ours has the significant advantage that breath-to-breath registration of nitrogen exchange at the mouth allows correction of the oxygen consumption and carbon dioxide production for breath-by-breath changes in lung gas stores (*18*–*20*). Although the particular measurement head developed in this study would not be suitable for exercise testing because the maximum flow through the flow sensor is too low and the analyzer head has not been optimized to be small and lightweight, there is, in principle, no reason why these limitations could not be overcome.

In summary, in-airway molecular flow sensing, using laser absorption spectroscopy coupled with accurate flow measurement, has the potential to provide simple, accurate, and noninvasive measurements of oxygen consumption in anesthesia and critical care. This has not proved possible with other existing technologies for measuring respiratory gas exchange. The real utility of easy, reliable measurements of oxygen consumption cannot be known without extensive clinical study. The development of the current device renders such studies possible.

## MATERIALS AND METHODS

The MFS measurement head connects in series with the main airway of the patient as shown in Fig. 1. This head incorporates both the gas analysis system and the flow measurement module. The electronics module is connected to the measurement head through two hybrid (optical and electrical) cables. In turn, the electronics module is connected to a PC through a USB cable.

### Characterization of the spectroscopic gas analyzer

The core technology of the gas analyzer is tunable diode laser absorption spectroscopy. For CO_2_ and H_2_O, the lasers (VL-2004-1-ST-H4, Vertilas; NLK1E5EAA, NTT Electronics), together with their control circuits, were located within the electronics module, and their outputs were multiplexed and coupled to the head by a single optical fiber. For O_2_, the control circuits were located in the electronics module, but the laser itself (ULM763-01-TN-S46FTT, ULM Photonics) was incorporated into the measurement head. This technique of laser absorption spectroscopy allows rapid and accurate analysis across the whole of the respired gas flow over a wide range of concentration values. The key spectroscopic parameters of the lines chosen for analysis are given in table S1.

**Fig. 5:**
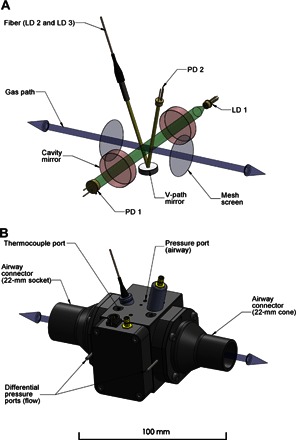
Design of the MFS measurement head. (**A**) Simplified diagram of the multichannel absorption spectrometer and the pneumotachograph contained within the measurement head. The bidirectional gas path (blue) is shown along with the two mesh screens (gray), across which the pressure drop related to the respiratory flow is measured. Radiation from the 764-nm diode laser (LD 1) used for probing oxygen is injected into an optical cavity constructed from a pair of highly reflective mirrors (red) and collected by a photodiode (PD 1) positioned along the optical axis (green). Two diode lasers, LD 2 and LD 3, located in the drive unit are used to probe for carbon dioxide and water vapor at 2004 and 1368 nm, respectively. Their outputs are spatially combined by a fiber-optic multiplexer located in the drive unit and are transmitted through a hybrid cable into the measurement cell. A fiber-optic collimator launches the radiation into the V path (yellow) and onto the photodiode (PD 2) via a concave mirror. (**B**) Three-dimensional CAD model of the measurement cell. The differential pressure ports located on the outer sides of the mesh screens are shown, together with the ports for the thermocouple probe and airway pressure sensor.

For CO_2_ and H_2_O, the transition strengths are similar and sufficiently large to be probed using direct absorption spectroscopy. In the instrument, we multiplexed the output of the two lasers, so they could share a common V-shaped optical path of ~5 cm across the measurement space. For O_2_, the transition strength is much weaker, and for this reason, we used the technique of off-axis, cavity-enhanced absorption spectroscopy, in which two highly reflective mirrors are used greatly to enhance the effective path length (*21*–*24*). Figure 5A shows the optical layout, and Fig. 5B shows a computer-aided design (CAD) model of the measurement cell.

For CO_2_ and H_2_O, the relationship between concentration and absorption is given directly by the Beer-Lambert law

$$I(\nu )=I_{0}(\nu )e^{-\sigma (\nu )CL}$$ (2)

where *I*(ν) and *I*_0_(ν) are the transmitted intensities at optical frequency ν in the presence and absence, over the path length *L*, of an absorber at concentration *C* and with an absorption cross-section of σ(ν). Because the ratio of *I*(ν) to *I*_0_(ν) depends only on species concentration, path length, and a molecule-specific physical parameter, the method is fundamentally calibration-free. For O_2_, the effect of concentration on absorption is given by a modified form of the Beer-Lambert law to account for the presence in the spectrometer of an optical cavity

$$\frac{I_{0}(\nu )-I(\nu )}{I(\nu )}=\frac{\sigma (\nu )CL}{1-R}$$ (3)

where the 1/(1 – *R*) term represents the path enhancement offered by a linear cavity with the geometric mean mirror reflectivity *R* (*25*). In contrast to CO_2_ and H_2_O, a calibration step using air (20.95% O_2_ in a dry sample) is required to determine 1/(1 – *R*).

**Fig. 6:**
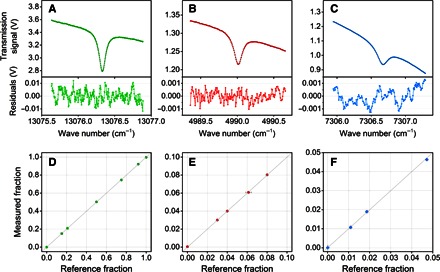
Characterization of the multichannel laser spectrometer. (**A** to **C**) Examples of transmission spectra associated with the spectral transitions of oxygen (green), carbon dioxide (red), and water vapor (blue). The spectrum measured by each spectrometric channel (colored symbols) is fitted to a predicted model (black line) by multiple linear regression analysis to retrieve the molecular concentration. The overall acquisition and processing time is within 10 ms. Residuals from the fitted model are shown in the subplots. (**D** to **F**) Calibration curves for the three spectrometric channels generated by a constant flow (50 liters/min) of pure gases and various calibration gas mixtures. The experimentally measured gas fractions are shown as symbols, with error bars in the *x* and *y* axes representing the absolute uncertainty (±1σ) in the composition of the gravimetrically prepared mixtures and in the output of the gas analyzer, respectively. For most data points, the error bars are contained within the size of the symbols. The line of identity is shown for reference.

For all three gases, the instrument is designed to provide an analysis every 10 ms. In practice, the lasers may be tuned across an absorption band to provide a spectrum more rapidly than this, and so two spectra are obtained and averaged within each 10-ms interval. Resulting spectra are shown in Fig. 6 (A to C). Note that the spectra are all for transitions between individual quantized energy levels of each gas and hence are highly selective: no other respiratory gases absorb in the spectral regions specific to each analyte. For each gas species, the concentration was estimated by regressing a theoretically predicted line shape onto the data. These line shapes were generated using the spectroscopic parameters reported in table S1, namely, the instantaneous sample temperature and pressure and the sample composition from the previous 10-ms time window, with line-broadening parameters taken from the literature (*26*). The lower panels of the plots in Fig. 6 show the residuals. The very small absolute magnitude of the residuals, together with the absence of systematic features, demonstrates the high quality of the fitting procedure.

The linearity of the O_2_ and CO_2_ analyses was explored by passing calibrated gas mixtures (certified accurate within ±1% of the specified gas fraction; BOC) through the cell. These covered a range of fractional contents for oxygen (0 to 100%), carbon dioxide (0 to 50%), and nitrogen (0 to 100%). For the water vapor analysis, linearity was checked by passing air through a respiratory gas humidifier (MR730, Fisher & Paykel) with the water bath set at various temperatures. The partial pressure of water vapor in each sample flowing through the cell was measured by a high-precision hygrometer that uses chilled mirror technology (Optidew, Michell Instruments), connected in series with the cell. High-purity grade nitrogen and oxygen (BOC) were used for investigating the lower limit of the dynamic range for all channels and the upper limit for the oxygen channel. The results (using 3-s averages for gas composition from the analyzer) are illustrated in Fig. 6 (D to F). More formally, we report in Table 1 the accuracy for each gas channel, defined as the maximum expected linearity error as a percentage of a notional full-scale output (FSO). The FSO was chosen as the highest percentage of the gas expected in the respired mixture under the experimental conditions (100% for O_2_, 10% for CO_2_, and 6% for H_2_O). For O_2_ and CO_2_, the accuracy levels achieved by the O_2_ and CO_2_ gas analyzers were well within the recommended (*2*) specifications (±1% FSO), despite the uncertainty of the composition of the gas reference mixtures being at a similar level to the specification. For water vapor, for which there is no recommended specification, there was a slightly poorer level of accuracy, but this may be attributed to limitations inherent in the reference analyzer or experimental procedure. Values for the precision of the 10-ms values returned by the analyzer, reported as a coefficient of variation (CV), are also shown in Table 1.

### Characterization of the flow meter

The flow measurement is accomplished with a modified, Lilly-style pneumotachograph, where the respiratory flow passes through a mesh and the flow rate is inferred from the pressure drop measured across the mesh. In the current system, the pressure drop is measured across a pair of meshes located at either end of the gas analysis space, as shown in Fig. 5, enabling the composition of the gas to be determined centrally within the flow measurement system.

Pneumotachograph systems are typically rather inaccurate, and a number of steps were taken to ensure that (i) a reproducible pressure drop could be recorded for a given flow and (ii) any given pressure drop could be accurately translated into a flow. To achieve a reproducible pressure drop, (i) helical flow conditioners, together with wire meshes, were used to ensure that any given flow impinges on the measurement mesh in a reproducible manner; (ii) multiple, small-bore holes distributed circumferentially around the airway were used to communicate with each pressure port of the differential pressure sensor to compensate for any unevenness of pressure around the circumference of the mesh; and (iii) an array of eight differential pressure sensors (HCLA, Sensortechnics) was used to reduce variability in the baseline differential pressure reading associated with zero flow.

If the flow is laminar, the pressure drop Δ*P* is linearly proportional to the flow $\dot{V}$ (the Poiseuille relationship), but this is far from true when the flow varies (and changes sign), as in the present application. We chose to model the pressure drop Δ*P* by an expression, which combines a “major head loss” term, which is linearly dependent on both viscosity and flow $\dot{V}$, and a “minor head loss” term, which is linearly dependent on density and proportional to the square of flow (*27*). Additional minor corrections for low flows under nonturbulent conditions and for the asymmetries of the flow system were introduced (*27*).

The basic relationship between pressure drop Δ*P* and flow is thus given by

$$\Delta P=\alpha \mu \dot{V}+\beta \rho {\dot{V}}^{2}$$ (4)

where $\dot{V}$ is the volumetric flow rate, μ is the instantaneous viscosity, ρ is the instantaneous density, and α and β are parameters of the pneumotachograph that are to be determined through calibration (*27*). A mechanical pump of fixed stroke volume was used for calibration purposes. Data were taken at different pump frequencies, with asymmetries between backward and forward flow and with change of calibrant gas from air to oxygen (thus, with changes in gas viscosity and density), all of which are situations that pertain to the applications of the device. The parameters α and β were determined through nonlinear regression to fit sets of integrated flows (volumes) to the known pump volume. During operation, μ and ρ were calculated every 10 ms from the simultaneously determined respired gas composition.

The data in Supplementary Materials show that the assumption of simple Poiseuille flow is inadequate, but the more complex relationship can predict the integrated volume flow from measurements of the pressure drop to a precision of better than 0.2%. This is more than an order of magnitude better than current guidance (see data in Table 1), but this accuracy is required for the successful measurement of ${\dot{V}}_{O_{2}}$ by direct integration of flow of oxygen into and out of the airway.

### Temperature and pressure management

Absolute pressure was sensed within the measurement space using a standard pressure sensor (HCA-BARO, Sensortechnics) connected to the airway pressure port, as shown in Fig. 5. To avoid condensation within the measurement space, the body of the chamber was gently warmed from 34° to 36°C. Temperature was monitored within the airway using four fine (26 μm) wire thermocouples positioned to give an average temperature for the overall gas flow, as determined following a simulation of gas flow and heat transfer within the measurement space.

## Supplementary Material

http://advances.sciencemag.org/cgi/content/full/2/8/e1600560/DC1

## SUPPLEMENTARY MATERIALS

Supplementary material for this article is available at http://advances.sciencemag.org/cgi/content/full/2/8/e1600560/DC1 Supplementary Methods Supplementary Results fig. S1. Calibration of the pneumotachograph. fig. S2. Effect of errors in the pneumotachograph calibration on gas exchange measurements. table S1. Summary of spectroscopic parameters.
