# Supplementary material for: In-airway molecular flow sensing: A new technology for continuous, noninvasive monitoring of oxygen consumption in critical care
Source: Sci Adv. 2016 Aug 10;2(8):e1600560. doi: 10.1126/sciadv.1600560 (PMC4980105; doi:10.1126/sciadv.1600560)
Supplement: http://advances.sciencemag.org/cgi/content/full/2/8/e1600560/DC1 [file supp_2_8_e1600560__index.html]

Science Advances | Science Advances

## Supplementary Materials

**This PDF file includes:**

- Supplementary Methods
- Supplementary Results
- fig. S1. Calibration of the pneumotachograph.
- fig. S2. Effect of errors in the pneumotachograph calibration on gas exchange measurements.
- table S1. Summary of spectroscopic parameters.

Download PDF

**Files in this Data Supplement:**

- Adobe PDF - 1600560\_SM.pdf
