# Supplementary material for: In-airway molecular flow sensing: A new technology for continuous, noninvasive monitoring of oxygen consumption in critical care
Source: Sci Adv. 2016 Aug 10;2(8):e1600560. doi: 10.1126/sciadv.1600560 (PMC4980105; doi:10.1126/sciadv.1600560)
Supplement: http://advances.sciencemag.org/cgi/content/full/2/8/e1600560/DC1 [file 1600560_SM.pdf]

Science Advances

AAAS

advances.sciencemag.org/cgi/content/full/2/8/e1600560/DC1

Supplementary Materials for

In-airway molecular flow sensing: A new technology for continuous, noninvasive monitoring of oxygen consumption in critical care

Luca Ciaffoni, David P. O'Neill, John H. Couper, Grant A. D. Ritchie, Gus Hancock, Peter A. Robbins

Published 10 August 2016, *Sci. Adv.* **2**, e1600560 (2016)
DOI: 10.1126/sciadv.1600560

This PDF file includes:

- Supplementary Methods
- Supplementary Results
- fig. S1. Calibration of the pneumotachograph.
- fig. S2. Effect of errors in the pneumotachograph calibration on gas exchange measurements.
- table S1. Summary of spectroscopic parameters.

Supplementary Methods

Calibration of the pneumotachograph

Figure S1 illustrates a typical set of experiments used to calibrate the pneumotachograph, using a pump with a stroke volume of 1.0 L operated under three different conditions. Shown in each case are the recorded pressure drop (left), the integrated volume that would result from an assumed linear relationship (Poiseuille law) between pressure drop and flow (middle) and the integrated volume that results from a more complex relationship between pressure and flow, equation (4), presented in the main text (right). The first experiment (fig. S1: A-C) involves a slow increase and then decrease in pump frequency. This illustrates that the integrated volume depends significantly on frequency if a simple linear relationship is assumed between pressure and flow, but that this is not the case for the more complex relationship. The second experiment (D-F) introduces a degree of asymmetry between inspiration and expiration for the mean flow rate, as inspiration is naturally of shorter duration that expiration. With this flow pattern, there is marked asymmetry for integrated volume with the simple linear relationship between pressure and flow, but again this is not present for the more complex relationship. The third experiment (G, H, J) is at a constant pump frequency, but switches the inspirate from air to oxygen around minute 2 (shown by the hatched region in the figure). Thus oxygen is inspired and a mixture of air (from the residual volume of the pump) with added oxygen is expired. This causes marked asymmetry between inspiration and expiration for the simple linear relationship between pressure and flow (H), but this does not occur with the more complex relationship where the calibration is dynamically compensated for changes in viscosity and density of gas as determined by the spectroscopic gas analysis system (J). In the figures the solid lines show error limits in the

cumulative volumes of ±0.2%, illustrating that the integration error between inspired and expired volume was well below our design target.

Supplementary Results

The effect of errors in the pneumotachograph calibration

Figure 3 in the main text shows the cumulative volumes of gas consumed/emitted as a function of time as calculated with the pressure/flow relationship of Equation 4 and using the calibrations of the type shown in fig. S1. Here we show the effect of changing the flows by a small amount, 3%, which is typical of the precision of standard flowmeter calibrations.

The left-most column shows net inspired volumes of each gas species through the O_2 wash-in experiment shown in Fig. 2. Number annotations show calculated time-averaged gas uptakes (negative values for net productions, measured in mL min^{-1}) over the duration of ~90 breaths as indicated by the vertical grey shading. The second column shows the same experiment but with an error in flow introduced such that inspired flows are 3% greater, and expired flows are 3% lower than best estimates. The right-most column shows the same experiment but with flow errors 3% lower for inspiration and 3% greater for expiration. Mean CO_2 production rates show changes of ~5%. Oxygen productions and nitrogen balances during air-breathing (first and last sections) change markedly. Oxygen consumptions during oxygen-breathing are drastically wrong, even showing an apparent rate of oxygen production.

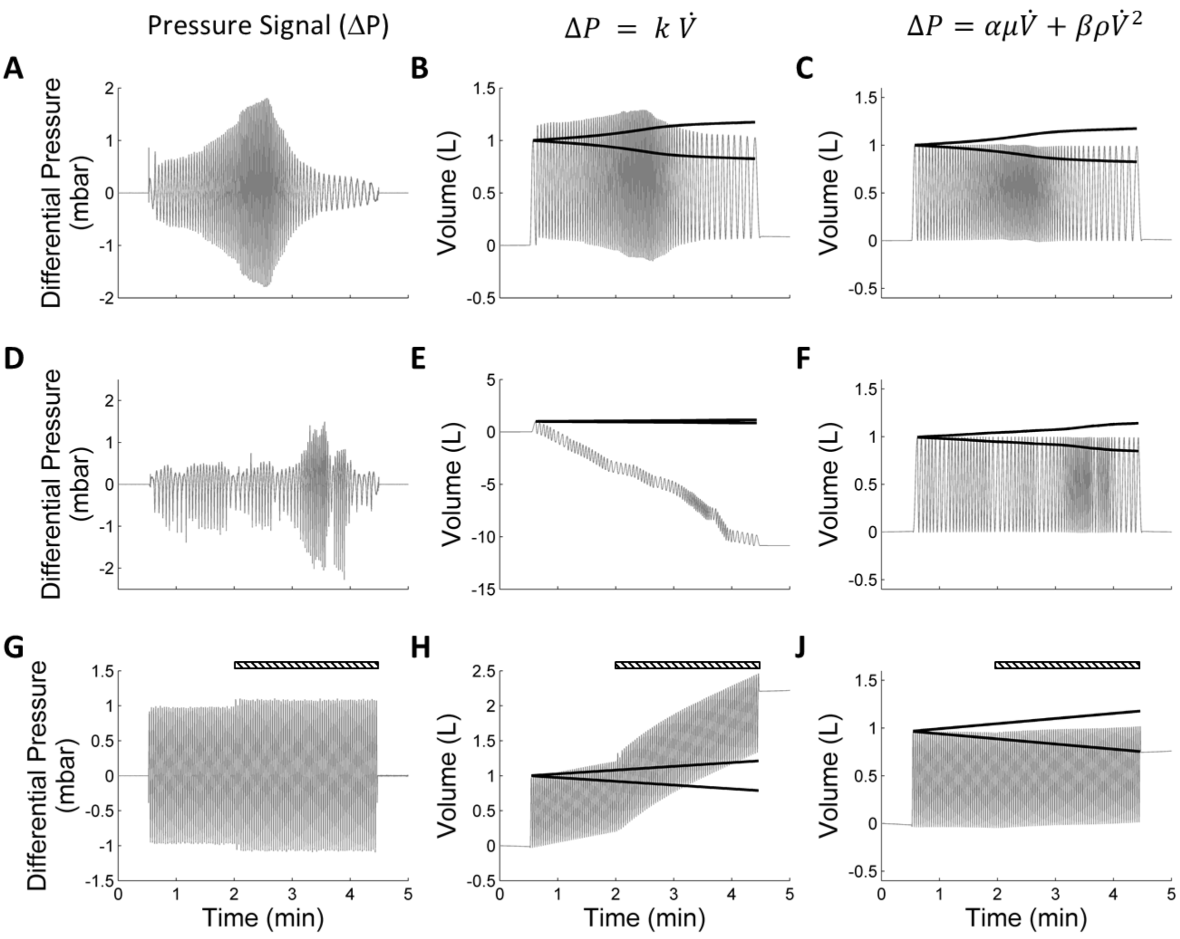

fig. S1. **Calibration of the pneumotachograph**. Experimental data for the pneumotachograph calibration, with the pump set at a constant stroke volume (1.0 L) and varying frequency (**A**), asymmetry (**D**) and gas composition (**G**). Also shown are the predicted flows using the simple Poiseuille law (**B**, **E**, **H**) and the more complex relationship given as Equation 4 in the main text (**C**, **F**, **J**). The solid lines indicate the range of possible values for tidal volume falling inside an error band of ± 0.2%. The hatched symbols (G, H, J) illustrate the period over which the inspirate was pure O_2.

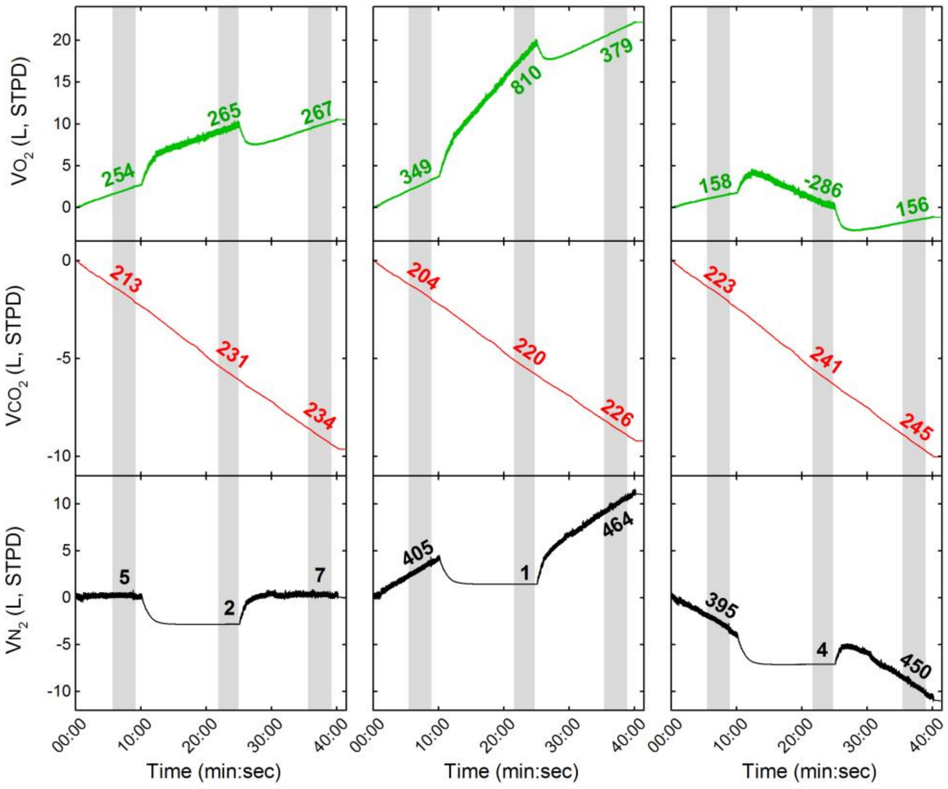

fig. S2. **Effect on gas exchange measurements of errors in the pneumotachograph calibration.** Effect on the calculated cumulative gas exchange volumes for errors of ±3% on the flow calibrations. The first column shows data analysed with the coefficients of equation 4 determined from the pump experiments shown in fig. S1. The second column shows the effect of increasing the expired flow and decreasing the inspired flow by 3%, with the third column reversing this flow asymmetry. The effect of the error is sufficiently large so as to produce an apparent net production of oxygen in the top right hand panel. Numbers given in the figure show the time average gas uptake/production in mL min^{-1} over the shaded regions.

table S1. Summary of the spectroscopic parameters. Summary of the parameters for the oxygen, carbon dioxide, and water vapor transitions employed in the analyser, as reported in the literature (*32*).

Molecule	O_2	CO_2	H_2O
Electronic transition	$b^1\Sigma_g^+ \leftarrow X^3\Sigma_g^-$	-	-
Vibrational band	$0 \leftarrow 0$	$201 \leftarrow 000$	$101 \leftarrow 000$
Rotational assignment [a]	${}^{P(13)}P(13)$	$R(18)$	$3_{13} \leftarrow 2_{12}$
Transition wavelength in vacuum (nm)	764.74	2004.02	1368.60
Transition strength at 296 K (cm^2 cm^{-1})	6.126×10^{-24}	1.287×10^{-21}	1.795×10^{-20}

[a] The rotational assignments are reported as ${}^{\Delta N(N'')}\Delta J\left(J''\right)$ for oxygen, $\Delta J\left(J''\right)$ for carbon dioxide, and $\left(J_{K_aK_c}\right)' \leftarrow \left(J_{K_aK_c}\right)''$ for water vapor, where the double prime ($''$) and single prime ($'$) symbols denote the lower and upper rotational energy states of the molecule, respectively.
